# The predictive value of pre-treatment MRI-based radiomics and clinical characteristics for medulloblastoma recurrence in pediatric patients

**DOI:** 10.3389/fneur.2025.1624819

**Published:** 2025-09-24

**Authors:** Huiwen Lu, Danzhu Li, Lixuan Huang, Zisan Zeng

**Affiliations:** Department of Radiology, The First Affiliated Hospital of Guangxi Medical University, Nanning, China

**Keywords:** medulloblastoma, recurrence, radiomics, MRI, pediatric patients

## Abstract

**Objective:**

The prognosis of medulloblastoma (MB) is extremely poor. This study aimed to develop a nomogram model for predicting the recurrence of MB in children by integrating pre-treatment magnetic resonance imaging radiomics and clinical characteristics.

**Methods:**

A retrospective analysis was conducted on 95 children with MB who were pathologically diagnosed with MB and underwent radical resection surgery. On the basis of recurrence status observed within the two-year post-treatment follow-up period, patients were categorized into recurrent and non-recurrent groups. The entire cohort was subsequently randomized into a training dataset and a test dataset using a 7:3 allocation ratio. Radiomic feature extraction was carried out utilizing the Feature Explorer Pro platform, with features derived from T1-weighted imaging (T1WI), T2-weighted imaging (T2WI), and contrast-enhanced T1-weighted imaging (T1WI_CE) sequences. The most significant features were selected using the Pearson correlation coefficient, analysis of variance (ANOVA), recursive feature elimination, and the Kruskal-Wallis test. A radiomics prediction model was developed using a support vector machine classifier. Logistic regression analysis was employed to identify the most valuable clinical characteristics, and they were used to develop a clinical model. The clinical and radiomics features were combined to develop a clinical-radiomics hybrid model, followed by establishing a nomogram. The predictive performance of each model was assessed using receiver operating characteristic curve analysis. The clinical utility of the model was evaluated via decision curve analysis (DCA) and calibration curves.

**Results:**

Two clinical characteristics and six radiomics features exhibiting the strongest associations with MB recurrence were selected to independently develop a hybrid model. The results showed that the hybrid model exhibited good predictive performance for MB recurrence in children. The AUC of the hybrid model reached 0.833 (95% confidence interval [CI], 0.730–0.937) in the training dataset and 0. 802 (95% CI, 0.635–0.970) in the test dataset, both of which exceeded the performance of the clinical model and the radiomics model. The calibration curve and DCA indicated that the nomogram possessed favorable clinical utility for predicting MB recurrence.

**Conclusion:**

The hybrid model, integrating pre-treatment MRI-based radiomics features and clinical characteristics, could effectively predict MB recurrence in pediatric patients.

## 1 Introduction

Medulloblastoma (MB) is the most frequent infratentorial malignant tumor in children, accounting for about 15–20% of children’s central nervous system (CNS) tumors (1). The current management of MB involves a risk-adapted multimodal strategy, comprising maximal surgical resection, postoperative craniospinal irradiation, and chemotherapy (2–4). After the aforementioned standardized treatment, approximately 70% of children achieve remission, and the 5-year survival rate can exceed 80% (5, 6). Nevertheless, relapse occurs in approximately 30% of cases, presenting as either localized or disseminated recurrence. Despite aggressive therapeutic interventions, such as secondary surgical resection, high-dose chemotherapy, intrathecal drug administration, re-irradiation, and anti-angiogenic therapy, the prognosis for recurrent MB remains unfavorable, with the long-term overall survival (OS) rate typically falling below 10% (6–9). Consequently, the early and precise detection of recurrence is crucial for promoting personalized treatment strategies in pediatric cases.

Risk stratification has long been recognized as a cornerstone in assessing treatment strategies and predicting prognosis in MB (10). Traditionally, risk classification has been based on clinical and pathological factors. However, due to the high degree of intratumoral heterogeneity, emerging evidence suggests that these conventional criteria may not fully capture the true prognostic risk in pediatric patients (11). For instance, some children classified as standard-risk experienced early relapse, while others did not receive sufficient treatment, leading to poor outcomes. In contrast, some high-risk patients underwent overly aggressive or unnecessary treatments, that may diminished quality of life, including endocrine and metabolic disorders, growth retardation, and other adverse clinical manifestations. This suggests that traditional risk stratification inadequately guides follow-up and personalized management. Recent studies have identified molecular subtypes as more robust predictors of prognosis. Among them, the WNT subgroup is associated with a favorable prognosis; the SHH and Group 4 subtypes correspond to intermediate outcomes, while Group 3 is linked to the poorest prognosis (12, 13). Nevertheless, the application of molecular subtyping remains limited, particularly in under-resourced settings, due to the high cost and technical complexity of genetic testing. As a result, there remains a need for an accessible, practical, and effective measure to predict MB recurrence.

MB is characterized by significant intratumoral heterogeneity, referring to variations in cellular phenotype, metabolism, and microenvironment across different tumor regions. Tumors exhibiting high heterogeneity tend to be more aggressive (14). MRI is a radiation-free imaging technique, playing significant roles in diagnosing MB, assessing residual tumor burden post-surgery, and evaluating tumor dissemination. Studies have demonstrated that MRI captures substantial latent information, capable of reflecting tumor heterogeneity, including gene expression levels, proliferative activity, and angiogenesis (15–17). Radiomics is a computational technique, which enables the extraction of large volumes of quantitative features from CT, MRI, or PET images and converts them into mineable, high-dimensional data (18). Radiomics has been widely utilized in CNS malignancies (19–21). For MB, radiomics has been mainly applied to the differential diagnosis of MB and the prediction of molecular subtypes (15, 22, 23). Furthermore, multimodal MRI radiomics has also been applied in MB. For example, Wang et al. developed a preoperative model for predicting the SHH and Group 4 subtypes based on T1WI, T2WI, T1C, FLAIR and ADC sequences (24). However, prognostic studies of MB remain limited, and they have mainly concentrated on long-term outcomes, such as OS, and only a few have addressed short-term outcome prediction.

This study investigated the predictive value of MRI based radiomics and clinical characteristics for identifying recurrence in pediatric MB, aiming to provide objective information for early detection of high-risk patients and the implementation of personalized therapeutic strategies.

## 2 Methods

### 2.1 Patients

A total of 95 pediatric patients who underwent radical resection surgery, and pathologically diagnosed MB from two center (center A, *n* = 59, center B, *n* = 36) between March 2011 and March 2023, were retrospectively analyzed. Basic clinical data were collected through the medical record system. The inclusion criteria were summarized as follows:(1) histopathological confirmation of MB; (2) age ranged from 0 to 18 years; (3) pre-treatment MRI performed within 2 weeks before surgical resection, including minimally the T1WI, T2WI, and T1WI_CE sequences; (4) regular follow-up for more than 2 years after surgical resection. The exclusion criteria were as follows: (1) poor-quality MR images (e.g., Severe motion artifacts); (2) presence of other CNS tumors; (3) patients who had received any anti-MB tumor treatment prior to this MRI examination; (4) the follow-up data was incomplete, or the follow-up period was less than 2 years.

According to the guidelines for response assessment in MB and leptomeningeal seeding tumors (25), patients were classified into the recurrent group if any of the following criteria were met within 2 years post-treatment: (1) ≥ 25% progression (compared to the smallest measurement recorded); (2) appearance of new disseminated lesions in the brain or in the spinal canal; (3) pathologically confirmed recurrence after secondary surgery; (4) conversion of cerebrospinal fluid cytology from negative to positive for tumor cells. Otherwise, they were classified into the non-recurrent group.

### 2.2 Clinical data collection

Clinical characteristics were collected from pediatric patients with MB, including sex, age, pathological type (classic, nodular or desmoplastic, anaplastic/large cell variants, extensive nodularity), tumor location (median if the vertical distance of the tumor center from the midline of the posterior cranial fossa was ≤1 cm, non-median if >1 cm), cystic degeneration/necrosis (yes/no), hemorrhage (yes/no), hydrocephalus (yes/no), degree of enhancement (mild/marked) and enhancement pattern (focal/incomplete/diffuse enhancement).

### 2.3 MR image acquisition

All patients underwent MRI within 2 weeks prior to treatment. The required imaging sequences included at least T1WI, T2WI, and T1WI_CE. Scans were performed using four different MRI systems: Siemens 3.0T, Canon 1.5T, Philips 3.0T, and GE 3.0T. These sequences share identical parameters when acquired using the same scanning machine. During T1-weighted enhanced imaging, gadolinium butanol was administered intravenously at a dosage of 0.1 mmol/kg body weight with an infusion rate of 2 mL/s. Detailed scanning parameters are presented in Table 1.

**Table 1:** Detailed parameters of magnetic resonance imaging.

Sequence	TR/TE (ms)	FOV (mm)	FA (°)	Slice thickness/gap (mm)	Voxel size (mm)
Siemens 3.0T					
T1WI	149/2.5	256 × 208	70	5/1	0.8 × 0.8 × 5.0
T2WI	4210/93	256 × 208	150	5/1	0.8 × 0.8 × 5.0
T1CE	467/2.5	256 × 208	70	5/1	0.9 × 0.9 × 5.0
Canon 1.5T					
T1WI	2100/17	288 × 224	90	6/1	0.5 × 0.5 × 6.0
T2WI	5320/119	288 × 224	90	6/1	0.5 × 0.5 × 6.0
T1CE	447.4/5.5	288 × 224	90	6/1	0.5 × 0.5 × 6.0
Philips 3.0T					
T1WI	2000/20	256 × 207	90	6/1	0.9 × 1.1 × 6.0
T2WI	2600/80	256 × 207	90	6/1	0.9 × 1.1 × 6.0
T1CE	2000/20	256 × 207	90	6/1	0.9 × 1.1 × 6.0
GE 3.0T					
T1WI	125.1/1.6	320 × 256	90	6/1	0.8 × 0.9 × 6.0
T2WI	6239/130	320 × 256	90	6/1	0.8 × 0.9 × 6.0
T1CE	162.4/1.6	320 × 256	90	6/1	0.8 × 0.9 × 6.0

### 2.4 Tumor segmentation and feature extraction

Tumor segmentation was performed using ITK-SNAP 4.2.2 software^1^. Axial T1WI, T2WI, and T1WI_CE sequences were imported into the software. A radiologist with 2 years of experience in neuroimaging manually delineated the ROI on each slice, carefully avoiding peritumoral edema and adjacent vasculature. The software subsequently generated the volume of interest. Segmentation results were reviewed and confirmed by another radiologist with over 20 years of experience. Both radiologists were blinded to patients’ information and recurrence status throughout the process. Radiomics feature extraction was conducted using Feature Explorer Pro (FAE, v0.5.13) in Python (3.7.6) (26). The process of feature extraction in this study followed the Image Biomarker Standardization Initiative (IBSI). Firstly, we performed N4 bias field correction on all the MRI images to correct the influence caused by the non-uniformity of the magnetic field. To unify the imaging differences among various MR devices, the voxel intensity values of all MR images were normalized to a range of [0, 1] using min-max normalization prior to feature extraction. This normalization process enhanced the comparability of the imaging data and laid a solid foundation for subsequent quantitative analysis. Then, the MR images were resampled to a uniform voxel size of 1 × 1 × 1 mm^3^. We imported the three sequences of all patients into the FAE software in sequence. After performing image preprocessing, we extracted radiomics features from each sequence in sequence. The feature types included First Order, Shape, and gray-level co-occurrence matrix (GLCM) features. The GLCM was configured with the following parameters: a quantization of 32 gray levels, a pixel distance of 1, and symmetric mode. After the feature extraction of each sequence was completed, a radiomics feature matrix was generated. Finally, the radiomics feature matrices of the three sequences were merged. Radiomics features with ICC ≥ 0.75 were retained and those with ICC < 0.75 were excluded. Furthermore, we applied the ComBat algorithm to eliminate the differences among various MR devices and different field strengths. Finally, the Dice coefficient was calculated to assess interobserver variability between the two radiologists, yielding a value of approximately 0.92, indicative of good agreement.

### 2.5 Feature selection

Ultimately, 168 radiomics features were extracted from the three sequences. A total of 56 features were extracted from each sequence, comprising 18 first-order features, 24 texture features, and 14 shape features. Subsequently, the entire cohort was randomized into a training dataset (*n* = 67, positive/negative = 27/40) and a test dataset (*n* = 28, positive/negative = 11/17). Radiomics model development was performed using pipelines developed in the FAE software. Firstly, up-sampling was performed in the training dataset by randomly duplicating cases until a balanced sample distribution was achieved. The up-sampling method was strictly confined to the training set and was not applied to the test dataset. In addition, normalization was applied to the feature matrix using Z-score and mean normalization. Due to the high-dimensional of the feature space, feature similarity was evaluated by computing the Pearson correlation coefficient (PCC) between each pair of features. Feature pairs exhibiting a PCC greater than 0.99 were excluded to minimize multicollinearity and enhance model robustness. This procedure effectively reduced the dimensionality of the feature space while maintaining feature independence.

Prior to model establishment, three feature selectors were employed: analysis of variance (ANOVA), recursive feature elimination (RFE), and the Kruskal-Wallis (KW) test. All three feature selection techniques were implemented in parallel. Support vector machine (SVM) was adopted as the classification algorithm due to its robustness and capability to project features into a higher-dimensional space for optimal label separation. Various combinations of feature selection methods and classifiers were compared, and the optimal model was identified based on the area under the curve (AUC). The hyperparameters were determined through 5-fold cross-validation. Among the 67 cases in the training dataset, four-fifths of the samples were utilized for model training in each iteration, while one-fifth were used for the validation. A 5-fold cross-validation procedure was implemented, resulting in 268 cases in the cross-validation training dataset (cv-train) and 67 cases in the validation dataset (cv-val). All samples from the training dataset were also utilized for model development and subsequently evaluated on an independent test dataset.

Finally, the radiomics model that combined ANOVA-based feature selection with a SVM classifier achieved the highest AUC. A total of six radiomics features were selected to develop the final radiomics model for predicting MB recurrence.

### 2.6 Model development

**Figure 1:**
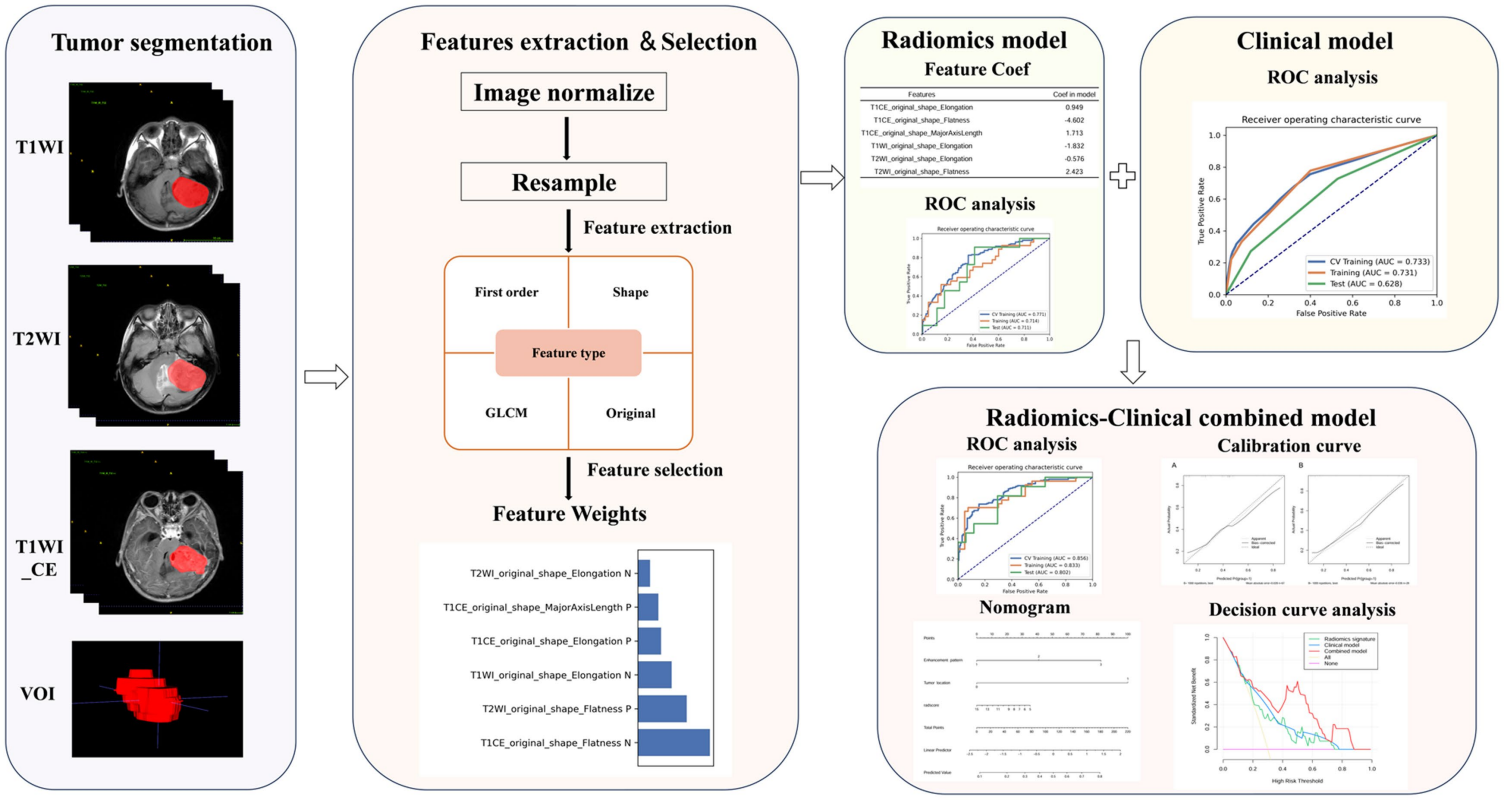
The study flowchart for predicting MB recurrence.

In the training dataset, the selected radiomics signatures were employed to calculate Rad-score. The clinical model was developed subsequently based on the selected clinical characteristics. The hybrid model was stored in the pickle serialization format of Python, enhancing its reproducibility and practical applicability in clinical settings. The selected significant clinical variables were combined with the Rad-score to develop a hybrid model using logistic regression analysis and then visualized as a nomogram, aiming to facilitate clinical application. Receiver operating characteristic (ROC) curves were plotted for all three models, and the AUC was calculated to evaluate their predictive performance. The 95% CIs were estimated using bootstrap resampling with 1,000 iterations. The DeLong test was applied to conduct a statistical comparison of the areas under the ROC curves. The Hosmer-Lemeshow test was employed to plot the calibration curve. Decision curve analysis (DCA) was employed to evaluate the net clinical benefit of each model in predicting recurrence. The overall methodology of the study is illustrated in Figure 1.

### 2.7 Data analysis

The data analysis in this study was conducted using R-studio 4.4.2 and SPSS 26.0 software. The differences of continuous variables were compared using *t*-test. Categorical variables were analyzed using either Chi-square test or Fisher’s exact test to assess intergroup differences. A *p*-value <0.05 indicated statistical significance.

## 3 Results

### 3.1 Clinical characteristics and conventional imaging features

A total of 95 children with MB were involved, including 38 cases in the recurrence group (28 men and 10women, with an average age of 8.3 ± 3.5 years) and 57 cases in the non-recurrence group (47 men and 10women, with an average age of 9.1 ± 4.1 years). The results of univariate analysis revealed that there were significant differences in three characteristics, including degree of enhancement, enhancement pattern and tumor location (*p* < 0.05, Table 2). Multivariate logistic regression analysis indicated that enhancement pattern and tumor location emerged as independent prognostic factors (*p*-values = 0.033 and 0.015 respectively).

**Table 2:** Univariate analysis of clinical characteristics and conventional imaging features.

Variable	Recurrence group (*n* = 38)	Non-recurrence group (*n* = 57)	Total (*n* = 95)	Statistical results (*χ*^2^/*t*)	*P*-value
Sex				*χ*^2^ = 1.056	0.304
Male	28 (73.7)	47 (82.5)	75 (78.9)		
Female	10 (26.3)	10 (17.5)	20 (21.1)		
Age (years), mean ± SD	8.3 ± 3.5	9.1 ± 4.1		*t* = 0.921	0.359
Pathological type				*χ*^2^ = 1.080	0.859
Classic	26 (68.4)	43 (75.4)	69 (72.6)		
Nodular or desmoplastic	6 (15.8)	7 (12.3)	13 (13.7)		
Anaplastic/large cell variants	5 (13.2)	5 (8.8)	10 (10.5)		
Extensive nodularity	1 (2.6)	2 (3.5)	3 (3.2)		
Ki-67 index				*χ*^2^ = 0.176	0.675
≥50%	21 (55.3)	29 (50.9)	50 (52.6)		
<50%	17 (44.7)	28 (49.1)	45 (47.4)		
Tumor location				*χ*^2^ = 4.791	0.007
Median	26 (68.4)	52 (91.2)	72 (79.1)		
Non-median	12 (31.6)	5 (8.8)	19 (20.9)		
Cystic degeneration/necrosis				*χ*^2^ = 1.056	0.304
Yes	28 (73.7)	47 (82.5)	77 (84.6)		
No	10 (26.3)	10 (17.5)	14 (15.4)		
Hemorrhage				*χ*^2^ = 0.925	0.336
Yes	4 (10.5)	3 (5.3)	7 (7.4)		
No	34 (89.5)	54 (94.7)	88 (92.6)		
Hydrocephalus				*χ*^2^ = 0.000	1.000
Yes	36 (94.7)	55 (96.5)	91 (95.8)		
No	2 (5.3)	2 (3.5)	4 (4.2)		
Degree of enhancement				*χ*^2^ = 4.967	0.026
Mild	6 (15.8)	21 (36.8)	27 (28.4)		
Marked	32 (84.2)	36 (63.2)	68 (71.6)		
Enhancement pattern				*χ*^2^ = 7.600	0.022
Focal enhancement	6 (15.8)	13 (22.8)	19 (20.0)		
Incomplete enhancement	6 (15.8)	21 (36.8)	27 (28.4)		
Diffuse enhancement	26 (68.4)	23 (40.4)	49 (51.6)		

### 3.2 Radiomics signatures

**Figure 2:**
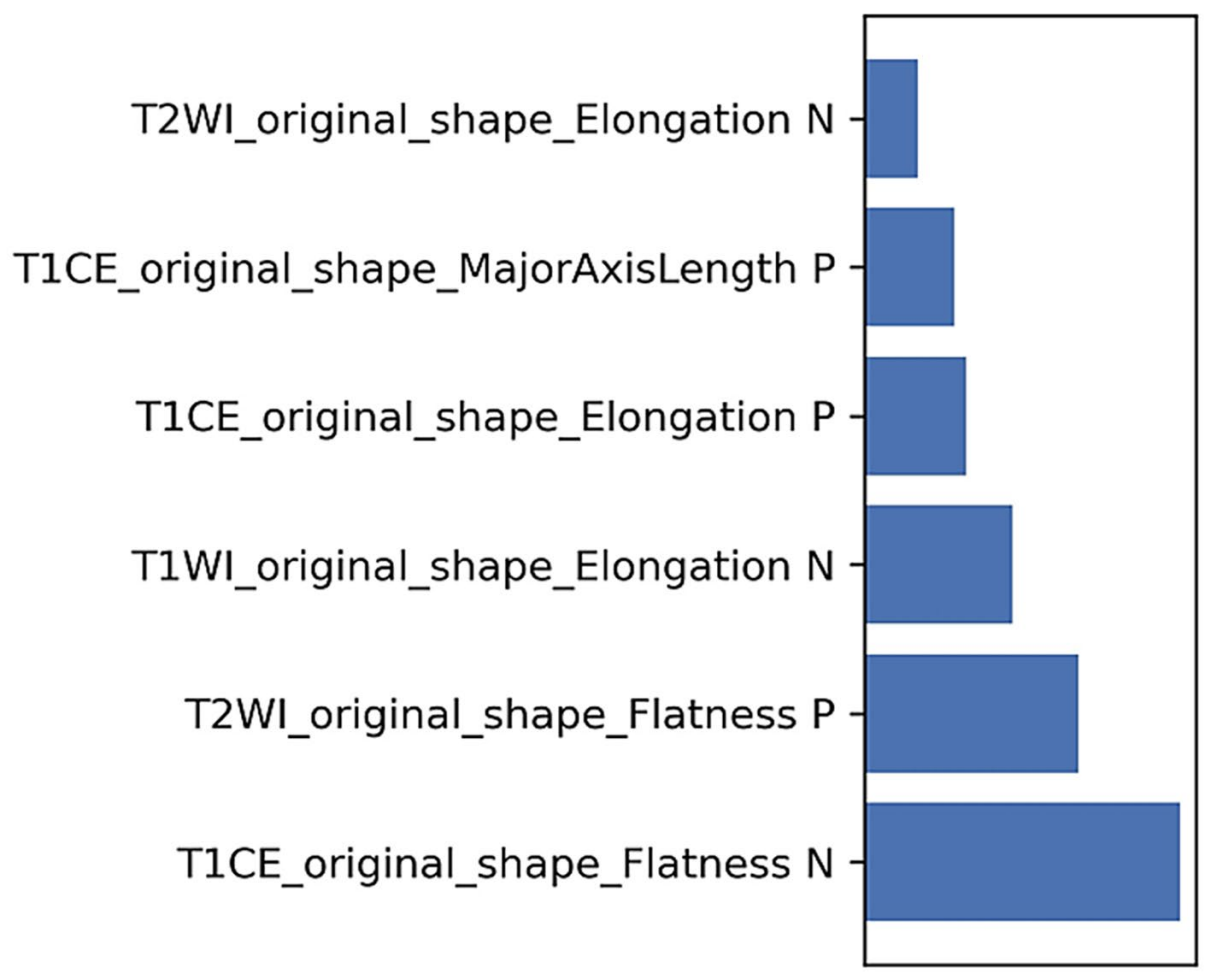
The weights of radiomics features.

Ultimately, 168 radiomics features were extracted for each patient. Six radiomics features were selected for developing the radiomics model (Table 3 and Figure 2), and the Rad-score was calculated as follows:


$$\begin{array}{c} \mathrm{Rad}-\text{score}=T1\mathrm{CE}\_\text{original}\_\text{shape}\_\text{Elongation}\times (0.282)\\ +T1\mathrm{CE}\_\text{original}\_\text{shape}\_\text{Flatness}\\ \times (-1.042)+T1\mathrm{CE}\_\text{original}\_\text{shape}\_\text{MajorAxisLength}\\ \times (0.328)+T1\mathrm{WI}\_\text{original}\_\text{shape}\_\text{Elongation}\\ \times (-0.438)+T2\mathrm{WI}\_\text{original}\_\text{shape}\_\text{Elongation}\\ \times (-0.168)+T2\mathrm{WI}\_\text{original}\_\text{shape}\_\text{Flatness}\\ \times (0.620)+0.034 \end{array}$$

**Table 3:** The coefficients of radiomics features.

Feature name	Coefficient in model
T1CE_original_shape_Elongation	0.282
T1CE_original_shape_Flatness	−1.042
T1CE_original_shape_MajorAxisLength	0.328
T1WI_original_shape_Elongation	−0.438
T2WI_original_shape_Elongation	−0.168
T2WI_original_shape_Flatness	0.620

### 3.3 Model performances

**Figure 3:**
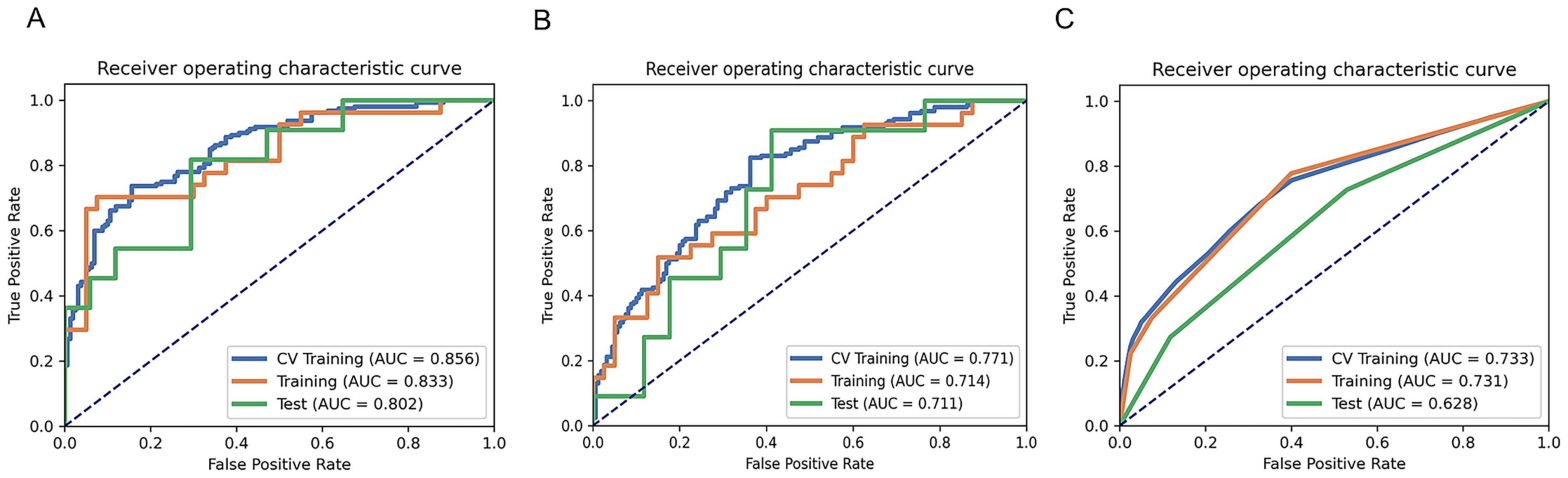
ROC curves of the hybrid model **(A)**, radiomics model **(B)**, and clinical model **(C)** for predicting the recurrence of MB.

**Figure 4:**
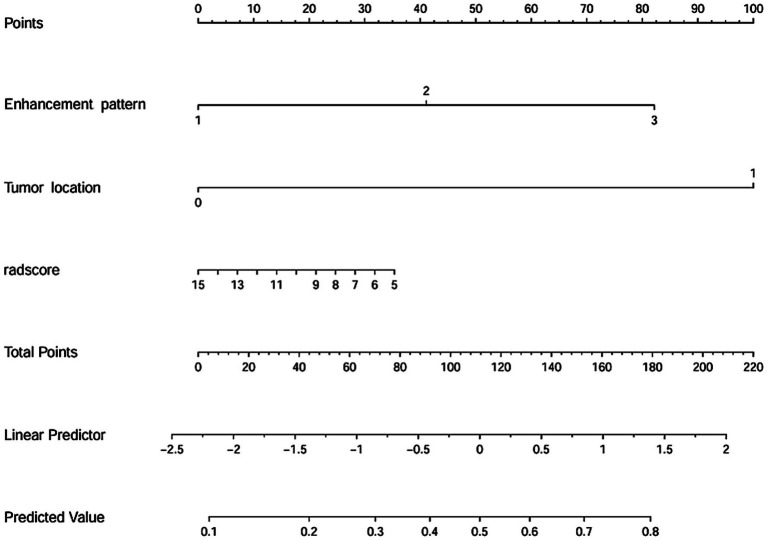
Nomogram for predicting the recurrence of MB.

**Figure 5:**
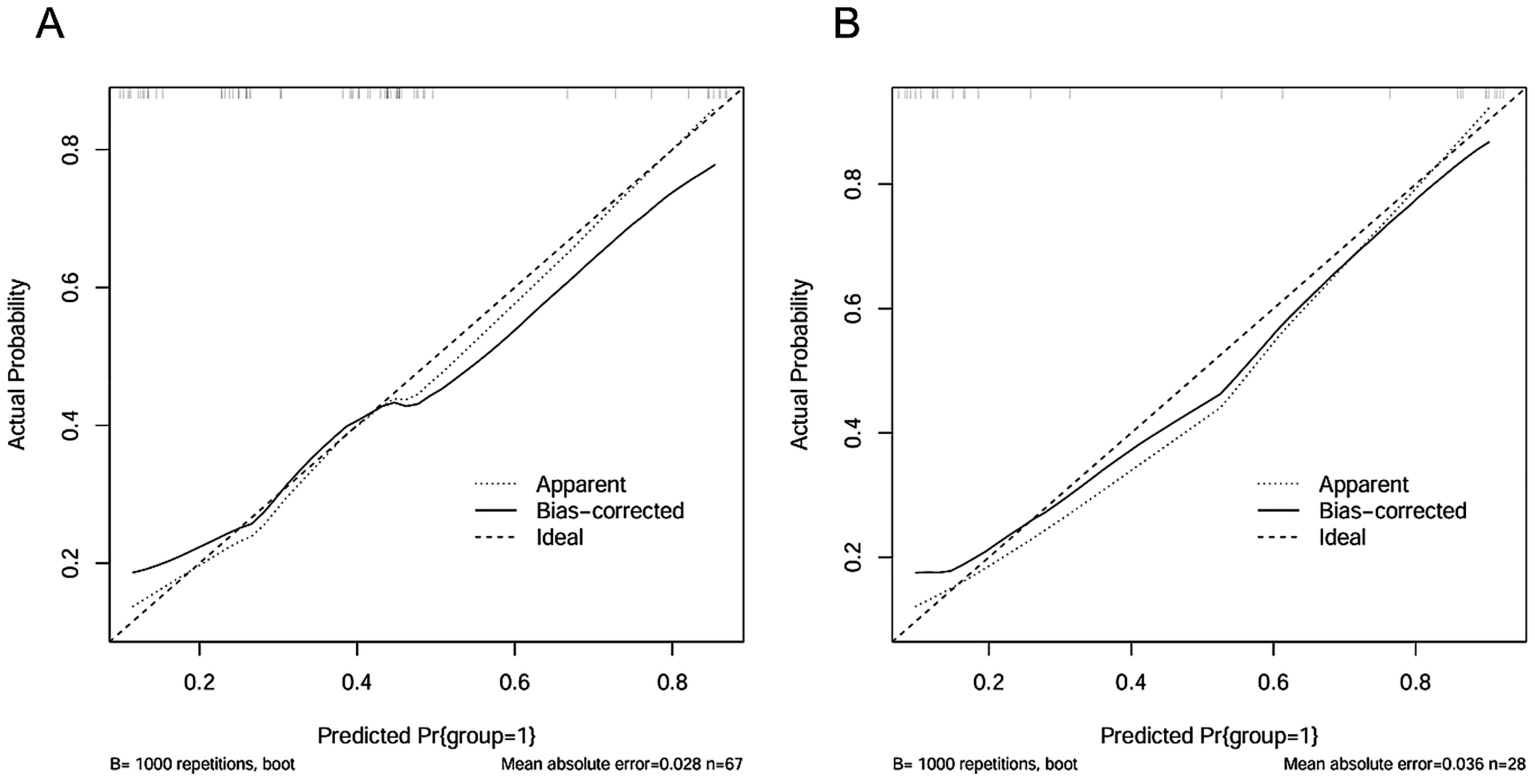
The calibration curves of the hybrid model in the training dataset **(A)** and the test dataset **(B)**.

**Figure 6:**
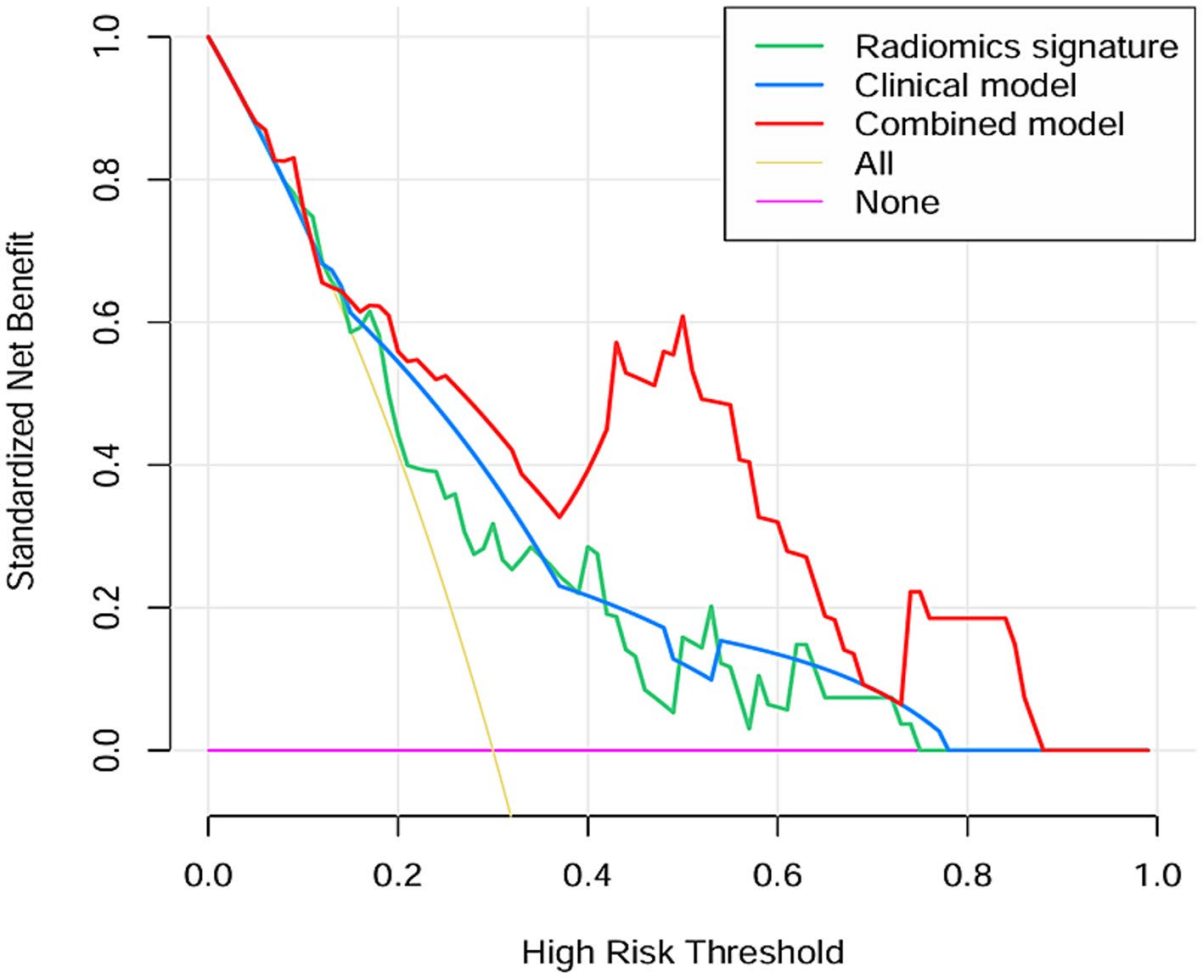
The DCA curve of the clinical model, radiomics model and hybrid model for predicting recurrence of MB.

Two clinical characteristics and six radiomics features were utilized to develop the clinical model, radiomics model, and hybrid model. The ROC curves are exhibited in Figure 3, and their performance metrics in predicting MB recurrence are detailed in Table 4. The results demonstrated that the hybrid model exhibited superior predictive performance. The AUC was 0.833 (95% CI: 0.730–0.937) in the training dataset and 0.802 (95% CI: 0.635–0.970) in the test dataset, both outperforming the clinical model (training dataset AUC = 0.731 [95% CI: 0.614–0.847]; test dataset AUC = 0.628 [95% CI: 0.425–0.832]) and the radiomics model (training dataset AUC = 0.714 [95% CI: 0.587–0.841]; test dataset AUC = 0.711 [95% CI: 0.514–0.909]). In the training dataset, the hybrid model achieved higher accuracy (0.821), sensitivity (0.780), and specificity (0.925) compared with the clinical model (0.672, 0.778, and 0.600, respectively) and the radiomics model (0.642, 0.704, and 0.600, respectively). These findings were similarly validated in the test dataset. Tumor location, enhancement pattern, and Rad-score were incorporated into a nomogram to facilitate clinical application (Figure 4). Calibration curves (Figure 5) demonstrated notable agreement between predicted and actual recurrence of MB. Moreover, DCA (Figure 6) indicated that when the threshold probability ranged from 0.15 to 0.70, the hybrid model provided greater net clinical benefit in predicting MB recurrence than the clinical or radiomics models alone.

**Table 4:** Performance parameters of the three models.

Model	AUC	95% CIs	Acc	Sen	Spe	PPV	NPV
Clinical model							
Training set	0.731	0.614–0.847	0.672	0.778	0.600	0.568	0.800
Test set	0.628	0.425–0.832	0.571	0.727	0.471	0.471	0.727
Radiomics model							
Training set	0.714	0.587–0.841	0.642	0.704	0.600	0.543	0.750
Test set	0.711	0.514–0.909	0.643	0.546	0.706	0.546	0.706
Hybrid model							
Training set	0.833	0.730–0.937	0.821	0.780	0.925	0.857	0.804
Test set	0.802	0.635–0.970	0.643	0.738	0.706	0.546	0.706

## 4 Discussion

This study investigated the predictive value of pre-treatment MRI-based radiomics and clinical characteristics for MB recurrence. The results indicated that the hybrid model developed by integrating tumor location, enhancement pattern, and Rad-score demonstrated excellent predictive performance in the recurrence of MB. This approach enables clinicians with a noninvasive, personalized method for the pre-treatment evaluation of the risk of MB recurrence, enabling early adjustment of treatment strategies, thereby improving pediatric prognosis positively.

The pathogenesis of MB remains elusive. Some studies have demonstrated that the occurrence of MB may be associated with genetics or genetic mutations. For instance, hereditary cancer susceptibility syndromes, such as Gorlin syndrome and Li-Fraumeni syndrome, are associated with an increased risk of MB (27, 28). Genetic mutations involved in these syndromes include suppressor of fused (SUFU), patched homolog 1 (PTCH1), adenomatous polyposis coli (APC), and tumor protein 53 (TP53) (27–29). However, most genetic susceptibility and mutation screening tests remain in the research and development phase and are not yet widely implemented in clinical practice. As a result, no definitive preventive or causative therapeutic strategies for MB are currently available. At present, the treatment of MB relies on a traditional multimodal approach. Nevertheless, due to the high degree of tumor heterogeneity, responses to radiotherapy and chemotherapy vary markedly among patients, leading to significant differences in clinical outcomes. Therefore, accurate evaluation of short-term prognosis and recurrence risk is of critical importance. Early identification of patients at high risk of recurrence allows for timely modification of treatment strategies, potentially reducing the negative consequences of under- or overtreatment, prolonging survival, improving prognosis, and enhancing overall quality of life.

Recurrent MB pediatric patients are confronted with significant health risks and complications, such as increased risk of tumor dissemination, significantly elevated difficulty in treatment, and severe impairment of neurocognitive function, imposing remarkable psychological and economic burdens on the affected children and their families. A retrospective study indicated that the 1-year OS rate of recurrent pediatric MB patients was 38.3% ± 4%, the 2-year OS rate was 16.9% ± 3.3%, and the 5-year OS rate was 12.4% ± 2.8% (30). These findings suggest that children with recurrent MB tend to have a poorer prognosis and lower survival rates. Recurrence has emerged as a critical determinant of MB outcomes, indicating that using recurrence rather than OS as the study endpoint may provide greater clinical value for pediatric patients. Given the limited efficacy of salvage therapies in treating recurrent MB, recent studies have emphasized the importance of recurrence prevention and early identification as the most promising strategies for improving outcomes in these cases (31). Consistently, the present study adopted recurrence as the primary endpoint. The predictive value of pre-treatment MRI-based radiomics features hybrid with clinical characteristics was evaluated, and a visualized nomogram model was developed to assist clinicians in performing comprehensive assessments and optimizing individualized treatment strategies for children with MB.

Several studies have identified various prognostic factors in pediatric patients with MB, including Chang stage, risk stratification, molecular subtype, tumor metastasis, postoperative radiotherapy and chemotherapy, as well as residual tumor volume (32–34). Although molecular subtyping is the most reliable predictor of MB prognosis, genetic testing was not included in this study due to its high cost and technical complexity, hindering its widespread clinical adoption. In the present study, tumor location, degree of enhancement and enhancement pattern demonstrated significant differences between the recurrent and non-recurrent groups, which align with results reported by Luo et al. (33) and Yan et al. (35). Tumor location has also shown to influence prognosis, possibly due to differences in the histological origins and preferential anatomical sites associated with specific molecular subtypes. For instance, WNT subtype typically arises in the brainstem or cerebellopontine angle, and SHH subtype predominantly occurs in the cerebellar hemispheres, while Group 3 and Group 4 subtypes are more frequently located in the midline posterior fossa (9, 36). In the present study, multivariate logistic regression analysis identified tumor location and enhancement pattern as independent risk factors for MB recurrence. The enhancement pattern is also correlated with tumor recurrence. This relationship may be explained by the observation that tumors exhibiting a more extensive enhancement area tend to have a richer blood supply, greater vascular leakage, and more pronounced disruption of the blood–brain barrier, coupled with a higher degree of malignancy, thereby demonstrating an increased propensity for recurrence (37).

Regarding radiomics-based prognostication in MB, the majority of existing studies have concentrated on long-term outcomes, such as overall survival (OS), while investigations targeting short-term endpoints, like recurrence remain comparatively limited. Luo et al. (33) developed a prognostic stratification model for MB. Their findings demonstrated that the radiomics nomogram can serve as a non-invasive method to predict pediatric MB prognosis, achieving AUC values of 0.926 and 0.835 in the training and validation datasets, respectively. However, their model did not incorporate relevant clinical risk variables, potentially limiting its predictive robustness. In contrast, a hybrid prognostic model was developed in the present study by integrating two key clinical parameters with six recurrence-associated radiomics features. Similarly, Liu et al. (34) developed a radiomics model for predicting progression-free survival in pediatric MB using features derived from T1WI and T1WI_CE sequences. Their results highlighted the advantage of integrating radiomics features with clinical factors, such as age and metastatic status over models based solely on clinical data. Nonetheless, their analysis was limited to two imaging modalities. In the current study, the feature extraction framework was extended by incorporating T2WI data, thereby enabling a more comprehensive radiomic characterization of tumor biology and heterogeneity.

In this study, six optimal radiomics features were identified to develop the model, including 6 shape features derived from T2WI and T1WI_CE sequences. Shape features describe the geometric morphology of the lesion, reflecting its three-dimensional spatial distribution and structural complexity. Irregular tumor shapes, including those with low sphericity and flatness, may be indicative of a poorer prognosis in MB. This could be attributed to the fact that aggressive tumors often exhibit high atypia, low cell differentiation, active mitotic activity, uncontrolled cell division, uneven local growth rate and angiogenesis, significant variations in blood supply, and inconsistent growth velocities in certain regions, leading to irregular morphologies. T2WI reflects the morphological features, signal characteristics, and internal cystic changes/necrosis of the tumors, reflecting the heterogeneity of tumors to some extent (38). In contrast, T1WI_CE reflects the tumor’s vascularity and disruption of the blood–brain barrier, offering a more comprehensive assessment of tumor heterogeneity (39). Furthermore, both T2WI and T1WI_CE are standard sequences used in the initial MRI evaluation of children with MB, and their widespread availability facilitates easy implementation in routine clinical practice. Notably, the application of multimodal MRI radiomics in MB is a direction that requires further research in the future. Moving forward, research efforts should integrate imaging sequences such as diffusion-weighted imaging (DWI), perfusion-weighted imaging (PWI), and magnetic resonance spectroscopy (MRS) to enhance the diagnostic accuracy and robustness of radiomics predictive models.

This study still exists several limitations. Firstly, molecular typing was not integrated as a predictive indicator in this study. Future research should investigate the impact of combining molecular typing with clinical and radiomics features on MB patients’ prognosis. Secondly, the sample size was limited, and no external data were included to validate the generalizability of the model. Therefore, larger sample size and prospective studies are required to advance this research field. Thirdly, this study only concentrated on the short-term prognosis of children within 2 years. Long-term follow-up studies and survival models (e.g., Cox, time-dependent AUC) are necessary to comprehensively assess such patients’ long-term prognosis in the future. Finally, it should be noted that, unlike adults, factors of pediatric growth may affect the radiomics analysis. Future research could consider setting a unified and smaller age range for the children participating in the study to mitigate potential confounding effects associated with growth-related factors.

## 5 Conclusion

The hybrid model combining radiomics and clinical variables could effectively predict the recurrence of pediatric MB. It may also serve as a non-invasive approach to help clinicians in the early identification of patients who are at high risk of recurrence and in formulating personalized treatment strategies, thereby improving the prognosis of children with MB.

## Data Availability

The raw data supporting the conclusions of this article will be made available by the authors, without undue reservation.

## Footnotes

^1^ http://www.itksnap.org/
